# Oral anticoagulation and self-management: analysis of the factors that determine the feasibility of using self-testing and self-management in primary care

**DOI:** 10.1186/1471-2261-13-59

**Published:** 2013-08-22

**Authors:** Eduardo Tamayo Aguirre, Itziar Vergara-Mitxeltorena, Edurne Uranga Saez del Burgo, Aitziber Ostiza-Irigoyen, Alejandro Garcia-Carro, Isabel Lopez-Fernandez, Arrate Galo-Anza

**Affiliations:** 1 Gros Health Centre, Donostia, Gipuzkoa, Spain; 2 Primary Care Research Unit-Gipuzkoa, Osakidetza, Spain; 3 Red de investigación en servicios de salud en enfermedades crónicas (REDISSEC), Centro de investigación en cronicidad Kronikgune, Osakidetza, Spain; 4 Pasai Donibane Health Centre, Gipuzkoa, Spain; 5 Usurbil Health Centre, Gipuzkoa, Spain; 6 Egia Health Centre, Donostia, Spain; 7 Lasarte Health Centre, Gipuzkoa, Spain

**Keywords:** Oral anticoagulants, Self-management, Determinants

## Abstract

**Background:**

The skills of patients on oral anticoagulants are critical for achieving good outcomes with this treatment. Self-management, or the capacity of patients to control their INR level and adjust their treatment, is an effective strategy of treatment. Capacity of patients to self manage is determined by a range of factors. The identification of these factors would improve the design of self management programmes and in turn increase the number of patients able to self-manage. The objective of our study is to identify those factors that determine the ability of patients on oral anticoagulant therapy to achieve self-management of their treatment.

**Design:**

This will be a three year quasi- experimental prospective study with a control group. 333 patients on anticoagulant therapy from five health centres of the Basque Health Service are to be followed up for a period of six months each after the intervention, to assess their ability to self-test and self-manage. The intervention will consist of a patient training programme involving the provision of information and practical training concerning their condition and its treatment, as well as how to use a portable blood coagulation monitoring device and adjust their anticoagulant dose.

**Discussion:**

The ease-of-use of this technique lead us to believe that self-management is feasible and will represent an innovative advance that should have a substantial impact on the quality of life of this patients and their families as well as on the health care provision systems.

**Trial registration:**

Osakidetza Protocol Record ISCIII-11/02285, Oral anticoagulation and self-management, ClinicalTrials.gov Identifier: NCT01878539

## Background

Oral anticoagulants (OACs) are increasingly widely used for the treatment of cardiovascular diseases, such as atrial fibrillation (currently its primary indication), valvular heart disease and venous thromboembolism [1]. Indeed, it in a relatively short period of time they have become the treatment of choice for the aforementioned conditions and are currently used in 1.7-2% of the population in Spain, reaching 7-8% in people aged 65 and [2] over. OACs are effective in preventing thromboembolic events, but they have a major adverse effect, namely an increased risk of bleeding [1].

While for OACs to be safe and effective international normalized ratio (INR) values should be maintained within narrow margins, measurements outside the normal range for their condition are common in people on OAC therapy. Indeed, internal assessments in centres in our region have estimated that 35-40% of INR measurements (around 19,000) are out of the normal range. Deviation from the therapeutic range has been associated with new cardiovascular events such as the development of thrombosis or bleeding in the case of low or high INR levels respectively [3].

Numerous factors have an impact on INR levels. Keeping these levels under control and stable is difficult and requires collaboration, learning and responsibility on the part of all those involved: general practitioners and nursing staff as well as patients themselves.

The progressive increase in the number of people on OACs has led to a corresponding increase in the resources dedicated to monitoring this treatment. Indeed, some hospital haematology services have been overwhelmed by situation, and in response to this several health regions in Spain have decided to transfer the follow-up and management of these patients to primary care. In primary care centres, the role has been taken on by general practitioners and primary care nursing staff who test INR levels and adjust doses as necessary (referring patients to the Haematology unit in just 6% of cases).

In this context, an increase in the number of patients able to self-monitor would have an impact at the organisational and financial level within the health system itself [4], both for hospital haematology services and primary care, given that management has been transferred to this level of care in many cases.

Moreover, there is an international consensus that self-monitoring [2] is a key element for improving the management of patients on OAC therapy. Internationally, several pilot studies [5] have been set up exploring patient self-testing (ability to use a coagulometer for measuring INR values) and self-management (ability to adjust doses according to the INR changes). These interventions, involving active selection of patients and subsequently providing them with training, have demonstrated that around 50% of patients are able to adequately monitor and manage their treatment [6,7]. Some interventions carried out in Spain, specifically in Andalucía, Cataluña, Castilla y León and Aragón (EviBased Nurs 2005: 8: 87 doi: http://dx.doi.org/10.1136/ebn.8.3.87) [4,7] have produced results confirming these findings. Notably, in some studies it has been found that patients who self-manage have lower rates of cardiovascular events and have significantly lower rates of thromboembolic and bleeding complications [5,8].

Many patients are able to monitor and even control factors that affect the therapeutic ranges and, in such cases, it is appropriate for them to take partial responsibility for managing their treatment. For this, however, patients must be trained how to use portable coagulometers and hence monitor their INR levels themselves, supported by health professionals [8-11].

Training programmes for self-management are effective in terms of enabling patients to acquire the necessary skills, though the level of success seems to depend on various patient-related factors. Given the proven benefits of self-management, it would be useful to properly characterise these factors and use this information to successfully increase the percentage of patients on OACs able to self-manage their own treatment.

## Design

This will be a quasi-experimental prospective study with a control group. Patients on anticoagulant therapy from five health centres of the Basque Health Service are to be followed up for a period of six months after the intervention, to assess their ability to self-monitor and self-manage.

### Hypothesis

An intervention program targeting patients and caregivers based on education about their condition and training in the use of portable coagulometer to self-monitor and training on the treatment adjustment to self manage, can be effective to enable patients in OACs to self manage as well as to identify determinant factors associated with the success and failure of the program.

### Primary objectives

1. To assess the ability of patients/caregivers to self manage OAC treatment (to test INR levels with a portable coagulometer and capillary whole blood from a finger prick and to adjuste OAC doses accordingly) 2. To evaluate the impact of a range of factors on the capacity of patients on OACs to self-monitor and self-manage.

### Secondary objectives

1. To compare the incidence of adverse effects in patients in the control and intervention groups 2. To compare the rate of in-range INR (i.e., levels within the therapeutic range) in patients in the control and intervention groups 3. To assess the impact on the quality of life of the self-management programme 4.To describe the attitudes and opinions of health professionals and patients with respect to the self-management programme.

## Methods

A pilot study has been conducted, over 33 patients, to identify potential determinant factors to be studied, to assess the methodology of the intervention and any technical problems related to the use coagulometers. The pilot study has already finished and has no lead to relevant changes in the main study protocol. Their results are being processed in order to be published.

Subsequently, a multi-centre, interventional study will be performed with a control group with an overall duration of three years. The target population for this study is patients on OAC therapy for any reason, aged above 16 years and under the care of participating health centres in an urban area. These health centres cover a population of over 200,000 and currently 1.7% of this population is on anticoagulants (approximately 3400 individuals).

Patients will be excluded from the study if they do not live in the area on a regular basis, have any serious diseases (including kidney or liver failure, or any terminal conditions), have been less than six months on OAC therapy or decline to participate. From the pool of elegible patients, the sample will be randomly selected, with a number of reserve subjects. These selected patients will be provided with information about the objectives and methodology of the study and asked to participate. Among these, some will be willing to be part of the intervention group and will be included in it, and some will not be interested in receive the intervention, and these will be considered for the comparison control group, both groups of patients should provide consent for their participation in the intervention and for their clinical records to be reviewed by the research team in the case of control group. This non random patient assignation method, is the reason of this study being defined as quasi-experimental and not a clinical trial.

The proposed sample size is 333 individuals. In order to derivate predictive models based on logistic regression models it is necessary to include at least ten final events for each of the independent variable included in the model (Laupacis A et al.; Vittinghoff E et al.). It is our intent to propose a model including less than ten independent variables, so, considering “successful self management” as our final event, 100 successful subjects will be necessary to guarantee a proper convergence of the model. Published studies [7] show that the success rate in this kind of self management programs stands around 50%, so we considered that in order to compare both groups, it would be necessary to have 100 successful patient and 100 non successful. Another study of Fitzmaurice [11] shows that a 40% of participants did not complete this type of program, so 333 subjects will be necessary in order to have the required sample size.

The intervention will consist of two sessions of participative training leaded by trained nurses. First, an educational workshop will be run with the goal of improving patient understanding of their condition and OAC treatment. Then, a second workshop will be run focused on training patients how to use a coagulometer and how to adjust their OAC doses, that is, the skills necessary to self-monitor and self-manage. In this second session, a coagulometer and a record sheet will be given to each patient as well as a telephone number to seek advice for any problems or questions that may arise during the follow-up period. The follow up period will last 6 months for each subject. Several intervention groups will be constituted and the intervention will be implemented consequently for each group, giving the project a total period of three years for its execution.

During this follow up period, patient will be provided free access to sanitary advice and a monthly follow-up visit will be scheduled with their assigned health professionals. Any reported incidents and weekly INR records kept by the patients will be registered, as will be details arised during the follow up visits.

Data will be collected on the use of the coagulometer, the INR measurements, the visits and any advice or support sought during the self-monitoring period. Other potentially relevant variables will also be analysed including age; use of multiple medications; willingness to take on self-management of their condition and treatment; social and cultural status; and presence of serious illnesses, conditions associated with tremor, multiple diseases, or blindness; as well as patient knowledge of their own disease and the reason for anticoagulation treatment. Particular attention will be paid to recording any thrombotic or bleeding episodes.

The main final event, “successful self management” will be defined as patients finishing the follow up period with less than 30% of INR determinations out of range and no thrombotic or bleeding episodes.

For self-monitoring, latest generation portable coagulometers will be used; these devices have improved compared to previous models and can be linked to a computer to download the data for processing.

Patients in the control group will receive regular care from their general practitioners. Data to calculate the rate of in-range INR (levels within the therapeutic range), and the incidence of adverse effects will be retrieved from the electronic clinical record for the six months follow-up period.

The attitude of health professionals, both doctors and nurses, with regards to whether they would advise self-monitoring and management in patients on OAC therapy will be analysed, using a questionnaire prior to the start of the study.

The project has been approved by the ethical committee “Comité Ético de Investigación Clínica del Área Sanitaria de Gipuzkoa” on November, the 23th 2011.

The project is funded by Carlos III Health Institute (a grant from the Spanish Health Research Fund, FIS (PI11/02285) and “Centro de Excelencia en Investigación en Cronicidad, Kronikgune” (Kronik 11/053).

## Discussion

### Potential impact of the results

In Spain, 1.7-2% of the general population and 7-8% of those above 65 years of age [1] are on OACs. Given that the mean age and life expectancy of the population is progressively increasing and that chronic disorders that require treatment with OACs are associated in most cases with age, the number of patients taking OACs in the near future can be expected to rise and to do so in proportion to the size of the over-70-year-old population.

The use of self-testing systems and self-management has hardly begun to be developed in our country [6], although it is estimated that about 50% of people on OACs are candidates for this approach. In the Basque Country (Spain), this figure corresponds to around 37,000 people (1.7% of the general population of around 2,178,000 people; Basque Institute of Statistics, March 2012).

At the same time, the latest information and communication technologies, the introduction in our health system of “Expert Patient”-type programmes and the ease-of-use of this technique lead us to believe that self-management is feasible and will represent an innovative advance that should have a substantial impact on the quality of life of this type of patients and their families as well as on the health care provision systems.

We are aware that other potential therapeutic alternatives are appearing, and new generation devices may be even easier to use, but they are still under research and additional data are necessary to assess their future introduction in primary care practice.

## Abbreviations

OACs: Oral anticoagulants; INR: Internacional normalizad ratio.

## Competing interest

The author(s) declare that they have no competing interests.

## Authors’ contributions

ET conceived the study, its design and coordination and drafted the manuscript. IV participated in the project design and manuscript draft and review. EU, AG, PS, AO, IL and AG are on charge of the patients recruitment, patients education and data collection. All authors read and approved the final manuscript.

## Pre-publication history

The pre-publication history for this paper can be accessed here:

http://www.biomedcentral.com/1471-2261/13/59/prepub
